# Fractional Telegrapher’s Equation under Resetting: Non-Equilibrium Stationary States and First-Passage Times

**DOI:** 10.3390/e26080665

**Published:** 2024-08-05

**Authors:** Katarzyna Górska, Francisco J. Sevilla, Guillermo Chacón-Acosta, Trifce Sandev

**Affiliations:** 1 Institute of Nuclear Physics, Polish Academy of Science, ul. Radzikowskiego 152, PL-31342 Kraków, Poland; katarzyna.gorska@ifj.edu.pl; 2 Instituto de Física, Universidad Nacional Autónoma de México, Apdo. Postal 20-364, Ciudad de México 01000, Mexico; fjsevilla@fisica.unam.mx; 3 Departamento de Matemáticas Aplicadas y Sistemas, Universidad Autónoma Metropolitana-Cuajimalpa, Vasco de Quiroga 4871, Santa Fe, Cuajimalpa, Ciudad de México 05348, Mexico; gchacon@cua.uam.mx; 4 Research Center for Computer Science and Information Technologies, Macedonian Academy of Sciences and Arts, Bul. Krste Misirkov 2, 1000 Skopje, Macedonia; 5 Institute of Physics, Faculty of Natural Sciences and Mathematics, Ss. Cyril and Methodius University, Arhimedova 3, 1000 Skopje, Macedonia; 6 Department of Physics, Korea University, Seoul 02841, Republic of Korea

**Keywords:** telegrapher’s equation, stochastic resetting, first-passage time

## Abstract

We consider two different time fractional telegrapher’s equations under stochastic resetting. Using the integral decomposition method, we found the probability density functions and the mean squared displacements. In the long-time limit, the system approaches non-equilibrium stationary states, while the mean squared displacement saturates due to the resetting mechanism. We also obtain the fractional telegraph process as a subordinated telegraph process by introducing operational time such that the physical time is considered as a Lévy stable process whose characteristic function is the Lévy stable distribution. We also analyzed the survival probability for the first-passage time problem and found the optimal resetting rate for which the corresponding mean first-passage time is minimal.

## 1. Introduction

Due to economic and military reasons in the XIX-th century, the rapid communication between the countries belonging to the British Empire was an important matter. To send a message from one part of the Empire to another was a true challenge, for its solution contributed to many famous physicists of that time, namely J. Maxwell, Lord Kelvin, and O. Heaviside. Nevertheless, only Heaviside solved this problem. He proposed the so-called telegrapher’s (telegraph) equation (TE) to describe the current propagation inside the telegrapher’s cable [1]. His equation is a hyperbolic equation which, in (1+1)-dimensional space, has the following form

(1) $$\partial_{t}^{2}p_{0}\left(x,t\right)+\tau^{-1}\partial_{t}p_{0}\left(x,t\right)=v^{2}\partial_{x}^{2}p_{0}\left(x,t\right),\;x\in R\;\mathrm{and}\;t\in R_{+},$$

where τ is a time parameter measured in seconds, and *v* is the propagation velocity measured in seconds/meter. The boundary conditions are set to zero at infinity, while the initial conditions are

(2) $$p_{0}\left(x,t=0\right)=\delta \left(x-x_{0}\right),\;\partial_{t}p_{0}\left(x,t\right){|}_{t=0}=0.$$

These boundary and initial conditions, allowed C. R. Cattaneo [2,3] and P. Vernotte [4] to employ Equation (1) to describe the heat transport. Thus, the physical interpretation of τ and *v* is changing. Here, τ modifies the Fourier law which connects the heat current j(x,t) and the temperature T(x,t) such that we have

$$j\left(x,t\right)+\tau^{-1}\partial_{t}j\left(x,t\right)∼-\partial_{x}T\left(x,t\right).$$

The parameter *v* is associated with heat conductivity. Recently, Equation (1) has been used to characterize the diffusion process with finite propagation velocity *v* [5], where $v=\sqrt{K/\tau }$, *K* is the diffusion coefficient, and the Fourier law is replaced by Fick’s law. In this case, the solution of the telegrapher’s equation is a probability density distribution (PDF) and, for arbitrary initial conditions, is presented in Ref. [6] (Equation (102) on p. 303 and/or [7] Equation (7.4.28)). This solution for the initial conditions given by Equation (2) reduces to the form

(3) $$\begin{array}{cc} p_{0}\left(x,t\right) & =\frac{1}{2}e^{-t/(2\tau )}\delta \left(vt-|x-x_{0}|\right)+\frac{1}{4v\tau }e^{-t/(2\tau )}\Theta \left(vt-|x-x_{0}|\right)\\ \; & \times \left[I_{0}\left(\frac{\sqrt{v^{2}t^{2}-{\left|x-x_{0}\right|}^{2}}}{2v\tau }\right)+\frac{vt}{\sqrt{v^{2}t^{2}-{\left|x-x_{0}\right|}^{2}}}I_{1}\left(\frac{\sqrt{v^{2}t^{2}-{\left|x-x_{0}\right|}^{2}}}{2v\tau }\right)\right], \end{array}$$

derived in Ref. [8]. We use the standard notation for which Θ(·) denotes the Heaviside step function, and $I_{\nu }\left(z\right)$ is the modified Bessel function of the first kind of order ν=0,1. The corresponding mean squared displacement (MSD) reads

(4) $${\langle x^{2}\left(t\right)\rangle }_{0}=\int_{R}x^{2}p_{0}\left(x,t\right)dx=2K\tau \left(\frac{t}{\tau }+e^{-t/\tau }-1\right),$$

which leads to the ballistic time dependence ${\langle x^{2}\left(t\right)\rangle }_{0}\approx v^{2}t^{2}$ in the short-time regime and to the linear time dependence ${\langle x^{2}\left(t\right)\rangle }_{0}\approx 2Kt$ in the long-time one.

Equation (1) characterizes the telegraph process whose corresponding Langevin equation reads

$$\dot{x}\left(t\right)=v\;\zeta \left(t\right),$$

where *v* denotes the constant particle speed, and ζ(t) is a stationary dichotomic Markov process that jumps between two states; ±1, with a mean rate ν [9,10,11,12] ($\tau =\frac{1}{2\nu }$, is a time scale that corresponds to the inverse mean sojourn time for each state). As shown in [13,14] subordinating this process by the Lévy process, we can find the fractional telegrapher’s equation of type I (abbreviated as FTE-I), namely

(5) τμDt2μCp1(x,t)+DtμCp1(x,t)=K∂x2p1(x,t),

where μ∈(0,1] and DtμCh(t) is the fractional derivative in the Caputo sense (see Appendix A). Equation (5) can be interpreted as an anomalous diffusion equation, which can be derived by using the continuity equation with an appropriate modified constitutive relation [15].

Despite the compelling generalization given by Equation (5), whose higher fractional derivative is always twice the lower one, it is possible to postulate different kinds of fractional telegrapher’s equations. For instance, we focus on one of these possibilities called type II (abbreviated as FTE-II), which has the form (this form corresponds to Equation (17) in Ref. [15])

(6) τDtμ+1Cp2(x,t)+DtμCp2(x,t)=K∂x2p2(x,t),

where μ∈(0,1]. In this case, the higher fractional derivative is always larger than 1. Equations (5) and (6) are completed with the same initial and boundary conditions (2) as for the telegrapher’s Equation (1). We can also find different fractional generalisations of the TE [5,10,15,16,17,18], including non-Markovian discrete time versions of the telegraph process [19], TE in random media [20,21,22], telegraph processes with random velocities [23], etc.

In this paper, we consider the stochastic resetting of the PDFs $p_{1}\left(x,t\right)$ and $p_{2}\left(x,t\right)$ that solve Equations (5) and (6), respectively. The stochastic dynamics of the particle, which are initially located at $x=x_{0}$ at t=0, under the effects of stochastic resetting, are described by the renewal equation [24]

(7) $$p_{r}\left(x,t\right)=\varrho \left(t\right)p_{j}\left(x,t\right)+\int_{0}^{t}\rho \left(t^{\prime }\right)p_{r}\left(x,t-t^{\prime }\right)dt^{\prime },$$

where j=0,1,2, indicates the probability density corresponding to the different telegrapher’s equations considered here; $\varrho \left(t\right)=\int_{t}^{\infty }\rho \left(t^{\prime }\right)dt^{\prime }$ is the probability that no renewal has taken place up to time *t*. We consider here the stochastic Poissonian resetting with probability $\rho \left(t\right)=re^{-rt}$ and from this, we have $\varrho \left(t\right)=e^{-rt}$, where *r* is the resetting rate. The renewal Equation (7) for Poissonian resetting has the form [24,25,26]:

(8) $$p_{r,j}\left(x,t\right)=e^{-rt}p_{j}\left(x,t\right)+\int_{0}^{t}re^{-rt^{\prime }}p_{j}\left(x,t^{\prime }\right)dt^{\prime },$$

which, in the Laplace space, reads

(9) $${\hat{p}}_{r,j}\left(x,s\right)=\frac{s+r}{s}{\hat{p}}_{j}\left(x,s+r\right),\;j=0,1,2.$$

Furthermore, we assume that the particle is instantaneously reset to the initial position. These assumptions offer the possibility of analytical calculations, as well a description of the experimental results for the mean-first passage time under stochastic resetting, by using holographic optical tweezers [27] or laser traps [28].

The paper is organized as follows. In Section 2, we consider the stochastic resetting of FTE-I. For that purpose, we find its solution in two ways based on integral decomposition techniques. This decomposition is rooted in recognizing the Brownian motion or the telegraph process. Next, we will reset the process described by FTE-I and calculate the first-passage time problem and survival probability. In Section 3, we repeat all procedures with the stochastic resetting for FTE-II. The paper is summarized in Section 4. The paper contains four Appendices.

## 2. FTE-I under Resetting

The PDF $p_{1}\left(x,t\right)$ can be found in two ways, which come from the Efros theorem (Appendix C) applied to $p_{1}\left(x,t\right)$. In the Laplace–Fourier (LF) space, it is written as

(10) $${\tilde{\hat{p}}}_{1}\left(\kappa ,s\right)=\frac{s^{-1}\left(\tau^{\mu }s^{2\mu }+s^{\mu }\right)}{\tau^{\mu }s^{2\mu }+s^{\mu }+K\kappa^{2}}e^{i\kappa x_{0}}=\frac{{\left[{\hat{M}}_{1}\left(s\right)\right]}^{-1}}{s{\left[{\hat{M}}_{1}\left(s\right)\right]}^{-1}+K\kappa^{2}}e^{i\kappa x_{0}},\;\mu \in \left(0,1\right).$$

Here,

(11) $${\hat{M}}_{1}\left(s\right)=\frac{\tau^{-\mu }s^{1-\mu }}{s^{\mu }+\tau^{-\mu }},$$

and s÷t and κ÷x are the Laplace and Fourier coordinates, respectively. The Efros theorem allows us to make the integral decomposition of Equation (10), for which we represent $p_{1}\left(x,t\right)$ as the integral $\int_{0}^{\infty }N\left(x,\xi \right)f_{1}\left(\xi ,t\right)d\xi$ with N(x,ξ) the Gaussian

(12) $$N\left(x,\xi \right)=\frac{1}{2\sqrt{\pi K\xi }}\;\exp\left[-\frac{{\left(x-x_{0}\right)}^{2}}{4K\xi }\right]$$

and

(13) $$f_{1}\left(\xi ,t\right)=L^{-1}\left\{{\left[{\hat{M}}_{1}\left(s\right)\right]}^{-1}e^{-\xi s/{\hat{M}}_{1}\left(s\right)};t\right\}.$$

The function $f_{1}\left(\xi ,t\right)$ for μ∈(0,1/2] is non-negative, such that it can be named the PDF of the leading process. Another possibility is to present $p_{1}\left(x,t\right)$ as the solution of the telegrapher’s equation $p_{0}\left(x,\xi \right)$ given by Equation (3) and $h\left(\xi ,t\right)=L^{-1}\left[{\left(\tau s\right)}^{\mu -1}\exp\left(-\xi \tau^{\mu -1}s^{\mu }\right);t\right]$ being for μ∈(0,1] the PDF of the leading process (for details, see Section 2.2). Hence, the solution obtained in this way is called the subordination approach. Both techniques in the presence of stochastic resetting will be described below, and we show that they lead to the same results.

### 2.1. Resetting of FTE-I—The First Possibility

Let us begin by examining the outcomes that can be derived from the second equality in Equation (10). In the short-time regime, s≫1/τ, we have that Equation (10) is approximated by

(14) $${\tilde{\hat{p}}}_{1}^{fWE}\left(\kappa ,s\right)∼\frac{\tau^{\mu }s^{2\mu -1}}{\tau^{\mu }s^{2\mu }+K\kappa^{2}}e^{i\kappa x_{0}},\;\mu \in \left(0,1\right),$$

which, after inverting the Laplace and Fourier transforms, we obtain the fractional wave equation (fWE)

(15) τμDt2μCp1fWE(x,t)=K∂x2p1fWE(x,t).

Transport in this regime is described by the fWE. In the opposite regime, i.e., in the long-time regime, s≪1/τ, Equation (10) is asymptotically approximated by

(16) $${\tilde{\hat{p}}}_{1}^{fDE}\left(\kappa ,s\right)∼\frac{s^{\mu -1}}{s^{\mu }+K\kappa^{2}}e^{i\kappa x_{0}},\;\mu \in \left(0,1\right),$$

which corresponds to the standard fractional diffusion equation (fDE) after inverting the Laplace and Fourier transforms, i.e.,

(17) DtμCp1fDE(x,t)=K∂x2p1fDE(x,t).

In the general case, upon performing the Fourier transform inversion of Equation (10), we can express it in Laplace space as:

(18) $$s{\hat{p}}_{1}\left(x,s\right)-\delta \left(x-x_{0}\right)=K\;{\hat{M}}_{1}\left(s\right)\;\partial_{x}^{2}{\hat{p}}_{1}\left(x,s\right),$$

which, after taking the inverse Laplace transform, can be written in *t*-space as the generalized diffusion equation

(19) $$\begin{array}{c} \begin{array}{cc} \partial_{t}p_{1}\left(x,t\right)=K\int_{0}^{t}M_{1}\left(t-t^{\prime }\right) & \partial_{x}^{2}p_{1}\left(x,t^{\prime }\right)dt^{\prime },\\ & M_{1}\left(t\right)=\tau^{-\mu }t^{2\mu -2}E_{\mu ,2\mu -1}\left(-{\left(t/\tau \right)}^{\mu }\right). \end{array} \end{array}$$

The series representation of the two-parameter Mittag–Leffler function $E_{\mu ,\nu }\left(z\right)$ is given by Equation (A1) for δ=1. After multiplying the last equation by $x^{2}$ and integrating over space we are led to first-order differential equations for the MSD, which can be integrated straightforwardly to give ${\langle x^{2}\left(t\right)\rangle }_{1}=2K\int_{0}^{t}\int_{0}^{t^{\prime }}M_{1}\left(s\right)ds\;dt^{\prime }$. We emphasize that the procedure of expressing the hyperbolic-like Equation (5) as the parabolic-like Equation (19) is only formal and it is possible only for the diffusion-like initial conditions (2). Formally, we can present the solution of Equation (19) as

(20) $$p_{1}\left(x,t\right)=\int_{0}^{\infty }N\left(x,u\right)f_{1}\left(u,t\right)du,$$

where N(x,u) and $f_{1}\left(u,t\right)$ are given by Equations (12) and (13), respectively. The same results can be obtained using Equation (A3) resulting from the Efros theorem in which $\hat{G}\left(s\right)={\left[{\hat{M}}_{1}\left(s\right)\right]}^{-1}$, $\hat{q}\left(s\right)=s/{\hat{M}}_{1}\left(s\right)$, and $\hat{g}\left(x,s\right)=\hat{N}\left(x,s\right)$. In the LF space, it figures as

(21) $${\hat{p}}_{1}\left(x,s\right)={\left[{\hat{M}}_{1}\left(s\right)\right]}^{-1}\;\hat{N}\left(x,s/{\hat{M}}_{1}\left(s\right)\right).$$

Note that $f_{1}\left(u,t\right)$ is a non-negative function for μ∈(0,1/2]. That comes from the Bernstein theorem [29] and the fact that ${\hat{f}}_{1}\left(u,s\right)$, for this range of μ, is a completely monotonic function (CMF), i.e., the non-negative function whose derivatives exist and alternate, see Appendix B. In consequence, we can say that $p_{1}\left(x,t\right)$, given by Equation (20) for μ∈(0,1/2] is a PDF expressed by the subordination approach, in which the leading process, described by $f_{1}\left(u,t\right)$, subordinates the parent process characterized by the normal distribution N(x,u) [13,14]. The function ${\hat{f}}_{1}\left(u,s\right)$ for μ∈(1/2,1] is not a CMF. Then, according to the Bernstein theorem, $f_{1}\left(u,t\right)$ is negative or contains negative parts. However, ${\hat{p}}_{1}\left(x,t\right)$ calculated from Equation (21) for μ∈(0,1] is a CMF, yielding that its inverse is non-negative and can be called a PDF.

**Figure 1:**
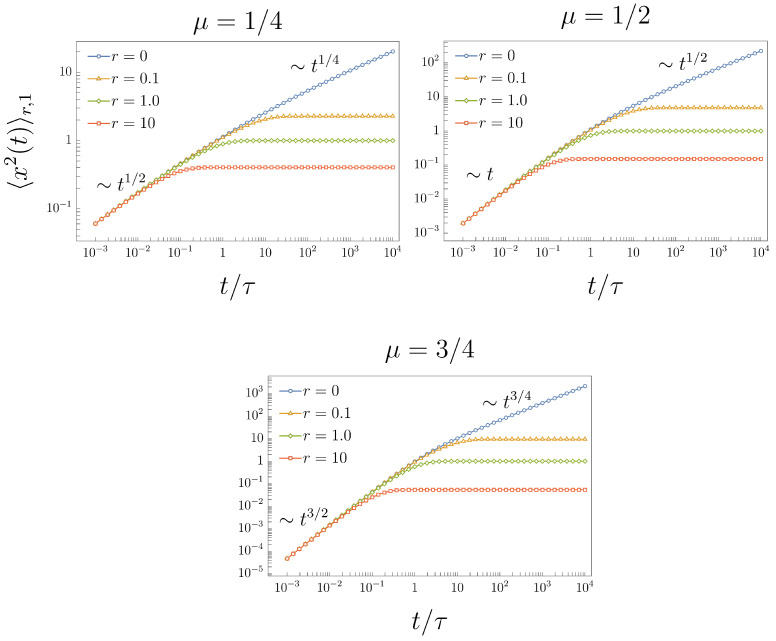
MSD (25) under the effects of stochastic resetting for different values of the resetting rate, r={0,0.1,1.0,10.0}. The case r=0 corresponds to the MSD (22).

The corresponding MSD ${\langle x^{2}\left(t\right)\rangle }_{1}$ can be expressed in terms of the MSD of the normal distribution ${\langle x^{2}\left(t\right)\rangle }_{N}=2Kt$, we have

$${\langle {\hat{x}}^{2}\left(s\right)\rangle }_{1}={\left[{\hat{M}}_{1}\left(s\right)\right]}^{-1}\;{\langle {\hat{x}}^{2}\left(s/{\hat{M}}_{1}\left(s\right)\right)\rangle }_{N}=\frac{2K}{\tau^{\mu }}\frac{s^{-\mu -1}}{s^{\mu }+\tau^{-\mu }},$$

from where, by inverse Laplace transform, one finds

(22) $$\begin{array}{c} {\langle x^{2}\left(t\right)\rangle }_{1}=2K\tau^{\mu }{\left(t/\tau \right)}^{2\mu }E_{\mu ,2\mu +1}\left(-{\left(t/\tau \right)}^{\mu }\right), \end{array}$$

which is a known result [5]. From (22), we have that ${\langle x^{2}\left(t\right)\rangle }_{1}\approx \left(2K/\tau^{\mu }\right)t^{2\mu }/\Gamma \left(2\mu +1\right)$ in the short-time regime, and ${\langle x^{2}\left(t\right)\rangle }_{1}∼2Kt^{\mu }/\Gamma \left(\mu +1\right)$ in the long-time regime; see Figure 1, curves marked with circle symbols for μ=1/4, 1/2, and 3/4.

If we reset $p_{1}\left(x,t\right)$ to the initial position according to the stochastic Poissonian resetting $\rho \left(\lambda \right)=re^{-r\lambda }$, where *r* is the resetting rate, then the corresponding $p_{r,1}\left(x,t|x_{0}\right)\equiv p_{r,1}\left(x,t\right)$ follows the renewal Equation (8) for j=1. In Laplace space, it reads Equation (9) for j=1, which, after employing Equation (21), gives

(23) $${\hat{p}}_{r,1}\left(x,s\right)=\frac{s+r}{s}\frac{1}{{\hat{M}}_{1}\left(s+r\right)}\hat{N}\left(x,\frac{s+r}{{\hat{M}}_{1}\left(s+r\right)}\right).$$

From here, we conclude that in the long-time limit, the system approaches the non-equilibrium stationary state (NESS) $p_{r,1}^{\mathrm{st}}\left(x\right)=\lim_{t\to \infty }p_{r,1}\left(x,t\right)=\lim_{s\to 0}s{\hat{p}}_{r,1}\left(x,s\right)=r{\hat{p}}_{1}\left(x,r\right)$, which is explicitly given by

(24) $$\begin{array}{c} \begin{array}{cc} p_{r,1}^{\mathrm{st}}\left(x\right) & =\frac{r}{{\hat{M}}_{1}\left(r\right)}\hat{N}\left(x,\frac{r}{{\hat{M}}_{1}\left(r\right)}\right)\\ & =\frac{1}{2}\sqrt{\frac{r^{\mu }\left(\tau^{\mu }r^{\mu }+1\right)}{K}}\exp\left(-\sqrt{\frac{r^{\mu }\left(\tau^{\mu }r^{\mu }+1\right)}{K}}\left|x-x_{0}\right|\right). \end{array} \end{array}$$

The NESS is given by the Laplace distribution with a stationary variance $\sigma_{1}^{\mathrm{st}}=K/\left[r^{\mu }\left(\tau^{\mu }r^{\mu }+1\right)\right]$ around the average value $x_{0}$. For μ=1, the NESS is analogous to Equation (27) of Ref. [8]. For τ=0, we recover the result for the NESS for an anomalous diffusion process under resetting [24,30,31,32,33], i.e., $p_{r,1}^{\mathrm{st}}\left(x\right)=r^{\mu /2}/\left(2\sqrt{K}\right)\exp\left(-\sqrt{r^{\mu }/K}\;\left|x-x_{0}\right|\right)$. We want to point out the exponential decay of the NESS tails, which is a characteristic induced by the stochastic Poissonian resetting process.

For the MSD in the case of resetting, we have

$$\begin{array}{cc} {\langle {\hat{x}}^{2}\left(s\right)\rangle }_{r,1} & =\frac{s+r}{s}{\langle {\hat{x}}^{2}\left(s+r\right)\rangle }_{1}\\ \; & =\frac{s+r}{s}\frac{1}{{\hat{M}}_{1}\left(s+r\right)}\;{\langle {\hat{x}}^{2}\left(\frac{s+r}{{\hat{M}}_{1}\left(s+r\right)}\right)\rangle }_{N}\\ \; & =2K\tau^{-\mu }s^{-1}\frac{{\left(s+r\right)}^{-\mu }}{{\left(s+r\right)}^{\mu }+\tau^{-\mu }}, \end{array}$$

from which we obtain

(25) $${\langle x^{2}\left(t\right)\rangle }_{r,1}=2K\tau^{\mu -1}\int_{0}^{t}e^{-rt^{\prime }}{\left(t^{\prime }/\tau \right)}^{2\mu -1}E_{\mu ,2\mu }\left(-{\left(t^{\prime }/\tau \right)}^{\mu }\right)dt^{\prime }.$$

In the short-time limit it behaves as ${\langle x^{2}\left(t\right)\rangle }_{r,1}∼t^{2\mu }$, and in the long-time limit it approaches the constant value,

(26) $${\langle x^{2}\left(t\right)\rangle }_{r,1}=\frac{2Kr^{-\mu }}{{\left(r\tau \right)}^{\mu }+1}.$$

The graphical representation of the MSD (25) for different values of μ and *r* is given in Figure 1, where we observe the asymptotic behavior of the MSD for short- and long-time limits.

### 2.2. Resetting of FTE-I—The Second Possibility

As mentioned in the Introduction, the FTE-I for μ∈(0,1] can be obtained from the telegraph process subordinated by Lévy noise. Here, we demonstrate this approach, starting from Langevin’s equations:

$$\dot{x}\left(u\right)=v\zeta \left(u\right)\;\mathrm{and}\;\dot{t}\left(u\right)=\xi \left(u\right),$$

where ζ(u) represents the same dichotomic noise as in the standard TE, and ξ(u) is a Lévy stable noise with Lévy index in Laplace space given by $\hat{\Psi }\left(s\right)=\tau^{\mu -1}s^{\mu }$, μ∈(0,1). Therefore, the process $t\left(u\right)=\int_{0}^{u}\xi \left(u^{\prime }\right)\;du^{\prime }$ is a stable Lévy motion with the characteristic function given by the stretched exponential function ${\hat{\Phi }}_{\mu }\left(u,s\right)=\exp\left(-u\tau^{\mu -1}s^{\mu }\right)$. Its inverse Laplace transform is denoted as $\Phi_{\mu }\left(u,t\right)$ and known as a one-sided Lévy stable distribution whose series form was found by H. Pollard in [34]. Its representation through the Fox *H* function was later found by R. Hilfer [35]. In [36], it was presented in the Meijer *G* form and the finite series of generalized hypergeometric functions. The corresponding PDF $p_{1}\left(x,t\right)$ of this subordinated telegraph process can be found from the subordination integral [13,14,37,38,39]

(27) $$p_{1}\left(x,t\right)=\int_{0}^{\infty }p_{0}\left(x,u\right)h\left(u,t\right)\;du,$$

where $h\left(u,t\right)=-\partial_{u}\langle \Theta \left(t-t\left(u\right)\right)\rangle$ is the subordination function which, in Laplace space, reads as

(28) $$\begin{array}{c} \begin{array}{cc} \hat{h}\left(u,s\right) & =-\frac{1}{s}\partial_{u}\langle \int_{0}^{\infty }\delta \left(t-t\left(u\right)\right)e^{-st}\;dt\rangle =-s^{-1}\partial_{u}\langle e^{-st(u)}\rangle \\ \; & =-s^{-1}\partial_{u}{\hat{\Phi }}_{\mu }\left(u,s\right)={\left(\tau s\right)}^{\mu -1}e^{-u\tau^{\mu -1}s^{\mu }}. \end{array} \end{array}$$

Inverting (28), we get the explicit time dependence of the subordination function

$$h\left(u,t\right)=L^{-1}\left[\hat{h}\left(u,s\right);t\right]=\frac{t}{\mu u}\Phi_{\mu }\left(u,t\right).$$

This subordination is also obtained from the first equality of Equation (10) by employing the Efros theorem in which $\hat{G}\left(s\right)={\left(\tau s\right)}^{\mu -1}$, $\hat{q}\left(s\right)=\tau^{\mu -1}s^{\mu }$, and $\hat{g}\left(x,s\right)={\hat{p}}_{0}\left(x,s\right)$ [13,14]. Then, from Equation (A3), we have

(29) $${\hat{p}}_{1}\left(x,s\right)={\left(\tau s\right)}^{\mu -1}{\hat{p}}_{0}\left(x,\tau^{\mu -1}s^{\mu }\right),$$

whose inverse Laplace transform gives Equation (27). From Equation (29), it follows that the MSD reads

$${\langle {\hat{x}}^{2}\left(s\right)\rangle }_{1}={\left(\tau s\right)}^{\mu -1}{\langle {\hat{x}}^{2}\left(\tau^{\mu -1}s^{\mu }\right)\rangle }_{0}=\frac{2K}{\tau^{\mu }}\frac{s^{-\mu -1}}{s^{\mu }+\tau^{-\mu }},$$

where $\langle x^{2}\left(t\right)\rangle$ is the MSD (4) for the standard TE. Therefore, we obtain the same MSD as in Equation (22), as expected. Thus, making the Poissonian resetting as in the previous subsection, we obtain Equation (25); in the long-time limit, this gives Equation (26). Moreover, NESS, given by Equation (23) for this kind of subordination, is also equal to Equation (24).

For μ=1, we recover the known results for the standard telegrapher’s equation with stochastic resetting [8].

### 2.3. First-Passage Time Problem

From Equations (18) and (9), we obtain

$$s{\hat{p}}_{r,1}\left(x,s\right)-\delta \left(x-x_{0}\right)=\frac{s}{s+r}{\hat{M}}_{1}\left(s+r\right)\partial_{x}^{2}{\hat{p}}_{r,1}\left(x,s\right),$$

where, by inverse Laplace transform, the generalized diffusion equation

$$\partial_{t}p_{r,1}\left(x,t\right)=K\int_{0}^{t}\left[M_{1}\left(t-t^{\prime }\right)-r{\bar{M}}_{1}\left(t-t^{\prime }\right)\right]\partial_{x}^{2}p_{r,1}\left(x,t^{\prime }\right)dt^{\prime },$$

is obtained, with memory function $M_{1}\left(t\right)-r{\bar{M}}_{1}\left(t\right)$, where

$${\bar{M}}_{1}\left(t\right)=e^{-rt}\int_{0}^{t}M_{1}\left(t^{\prime }\right)dt^{\prime }.$$

Let us write the corresponding backward equation for the survival probability $Q_{1}\left(x_{0},t\right)$, which will give the probability of the particle starting at $x_{0}>0$ to reach the target at the origin. Thus, we have

Dt2μCQ1(x0,t)+τ−μDtμCQ1(x0,t)=v2∂x02Q1(x0,t),

with initial conditions

$$Q_{1}\left(x_{0},0\right)=1,\;\partial_{t}Q_{1}\left(x_{0},t\right){|}_{t=0}=0,$$

and boundary conditions $Q_{1}\left(0,t\right)=0$ and $Q_{1}\left(\infty ,t\right)=0$. By Laplace transform, we find that

$${\hat{Q}}_{1}\left(x_{0},s\right)=\frac{1}{s}\left\{1-\exp\left(-\sqrt{\frac{\tau^{\mu }}{K}\left[s^{\mu }+\tau^{-\mu }\right]s^{\mu }}x_{0}\right)\right\}.$$

From here, we can calculate the first-passage time density $P_{1}\left(t\right)=-\frac{d}{dt}Q_{1}\left(x_{0},t\right)$, i.e., ${\hat{P}}_{1}\left(s\right)=1-s{\hat{Q}}_{1}\left(x_{0},s\right)$, see [40,41,42], which, in the Laplace space, reads

$${\hat{P}}_{1}\left(s\right)=\exp\left(-\sqrt{\frac{\tau^{\mu }}{K}\left[s^{\mu }+\tau^{-\mu }\right]s^{\mu }}x_{0}\right).$$

If we consider the process as a random search, then we can calculate the efficiency of the search, defined as the number of visited targets divided by the average number of steps needed. If there is a single target, it can be calculated as the inverse of the MFPT [43]

(30) $$\begin{array}{cc} E_{1}=\langle \frac{1}{t}\rangle = & \int_{0}^{\infty }\frac{P_{1}\left(t\right)}{t}dt=\int_{0}^{\infty }{\hat{P}}_{1}\left(s\right)\;ds\\ \; & =\frac{1}{2^{1/\mu }\mu }\Gamma \left(1+\frac{1}{2\mu }\right){\left(\frac{2\sqrt{K}}{x_{0}}\right)}^{1/\mu }\exp\left(-\frac{x_{0}}{2\sqrt{K\tau^{\mu }}}\right), \end{array}$$

whose derivation is presented in Appendix D.

If we further consider exponential resetting to the telegraph process, then for the survival probability, one finds [41,42]

$${\hat{Q}}_{1,r}\left(x_{0},s\right)=\frac{{\hat{Q}}_{1}\left(x_{0},s+r\right)}{1-r{\hat{Q}}_{1}\left(x_{0},s+r\right)},$$

from where we derive the MFPT

(31) $$\begin{array}{c} \begin{array}{cc} T_{1,r}\left(x_{0}\right) & =-\int_{0}^{\infty }t\;\left[\partial_{t}Q_{1,r}\left(x_{0},t\right)\right]\;dt=Q_{1,r}\left(x_{0},s=0\right)\\ & =\frac{1}{r}\left[\exp\left(\sqrt{\frac{r^{\mu }\left(\tau^{\mu }r^{\mu }+1\right)}{K}}x_{0}\right)-1\right]. \end{array} \end{array}$$

We see that the MFPT in the limits r→0 and r→∞ diverges, so there is an optimal resetting rate $r_{*}$ for which MFPT is minimal, i.e.,

$$\partial_{r}T_{1,r}\left(x_{0}\right){|}_{r=r_{*}}=0,$$

from where we have

(32) $$\begin{array}{c} \frac{\mu (1+2r_{*}^{\mu }\tau^{\mu })}{2(r_{*}^{\mu }\tau^{\mu }+1)}\sqrt{\frac{r_{*}^{\mu }\left(\tau^{\mu }r_{*}^{\mu }+1\right)}{K}}x_{0}=1-\exp\left(-\sqrt{\frac{r_{*}^{\mu }\left(\tau^{\mu }r_{*}^{\mu }+1\right)}{K}}x_{0}\right), \end{array}$$

i.e.,

$$1-e^{-\xi_{1}}=r_{*}\frac{d\xi_{1}}{dr_{*}},\;\xi_{1}=\sqrt{\frac{r_{*}^{\mu }\left(\tau^{\mu }r_{*}^{\mu }+1\right)}{K}}x_{0}.$$

For τ=0 it reduces to [44]

$$\frac{\mu }{2}\sqrt{\frac{r_{*}^{\mu }}{K}}x_{0}=1-\exp\left(-\sqrt{\frac{r_{*}^{\mu }}{K}}x_{0}\right).$$

For μ=1, we recover the MFPT for the telegraph process under resetting [8], see also [45],

$$T_{r}\left(x_{0}\right)=\frac{1}{r}\left[\exp\left(\sqrt{\frac{r(\tau r+1)}{K}}x_{0}\right)-1\right].$$

while for τ=0, the result for the subdiffusive search [44,46]

$$T_{r}\left(x_{0}\right)=\frac{1}{r}\left[\exp\left(\sqrt{\frac{r^{\mu }}{K}}x_{0}\right)-1\right],$$

and for τ=0 and μ=1, the Brownian search with [47]

$$T_{r}\left(x_{0}\right)=\frac{1}{r}\left[\exp\left(\sqrt{\frac{r}{K}}x_{0}\right)-1\right].$$

**Figure 2:**
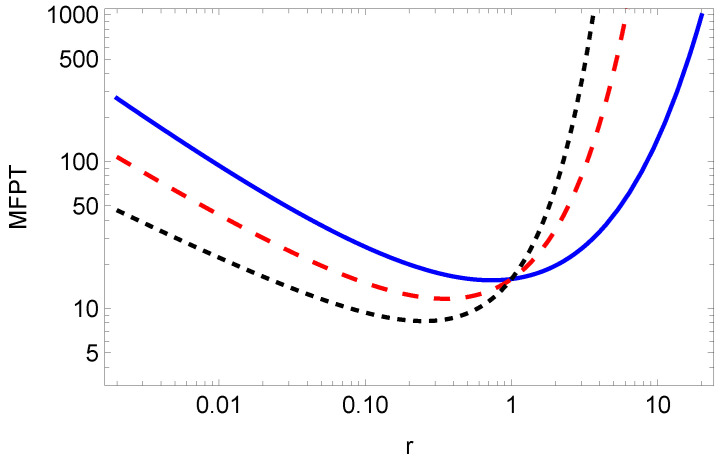
MFPT (31) for $x_{0}=2$, τ=1, K=1, μ=1/2 (blue solid line), μ=3/4 (red dashed line), μ=1 (black dotted line).

**Figure 3:**
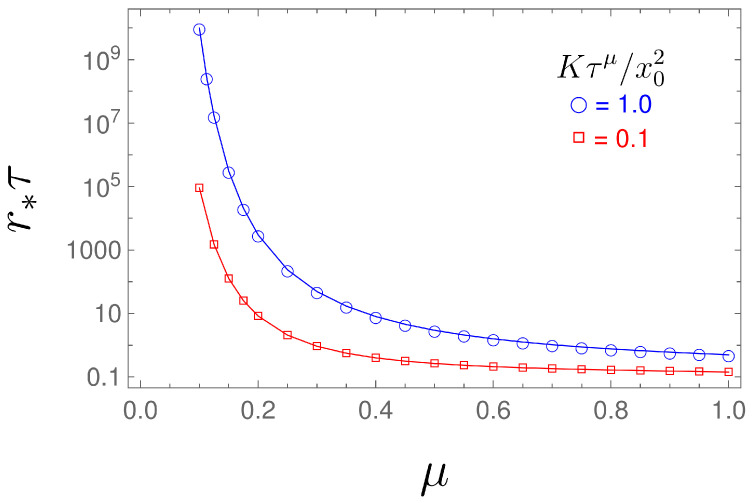
Optimal resetting rate $r_{*}$ versus μ, obtained by numerically solving Equation (32).

The dependence of the MFPT on the resetting rate *r* is shown in Figure 2, while the optimal resetting rate versus parameter μ is shown in Figure 3. From Equation (32), for a given exponent μ, we can numerically find the optimal value *r* for which the MFPT is minimum, see Figure 3. From Figure 2, we see that by increasing μ, the resetting rate $r_{*}$, for which the MFPT is minimum, decreases. Therefore, we need a higher resetting rate in order to reset the particle, which is stacked due to the long-tailed waiting time, given by the fractional exponent μ (the lower fractional exponent μ means a longer waiting time, and therefore, we need a higher resetting rate for the particle to reach the target in a shorter time).

## 3. FTE-II under Resetting

By the same methods used in the previous section, we have that the Laplace transform of Equation (6) looks analogous to Equation (12), but with the difference that instead of ${\hat{M}}_{1}\left(s\right)$, we have to use ${\hat{M}}_{2}\left(s\right)$ defined as

$${\hat{M}}_{2}\left(s\right)=\frac{1}{\tau }\frac{s^{1-\mu }}{s+\tau^{-1}}\;\mathrm{and}\;M_{2}\left(t\right)=L^{-1}\left[{\hat{M}}_{2}\left(s\right);t\right]=\frac{t^{\mu -1}}{\tau }E_{1,\mu }\left(-\frac{t}{\tau }\right).$$

Proceeding similarly as in the previous case of FTE-I to obtain Equation (18), we have that

(33) $$s{\hat{p}}_{2}\left(x,s\right)-\delta \left(x-x_{0}\right)=K{\hat{M}}_{2}\left(s\right)\partial_{x}^{2}{\hat{p}}_{2}\left(x,s\right).$$

In the short-time regime, we have that the memory function ${\hat{M}}_{2}\left(s\right)∼1/\left(s^{\mu }\tau \right)$; therefore, Laplace inversion of Equation (33) is possible since μ∈(0,1]. Thus, we have the fWE

(34) τDtμ+1Cp2fWE(x,t)=K∂x2p2fWE(x,t).

In the long-time regime ${\hat{M}}_{2}\left(s\right)∼s^{1-\mu }$ we recover the fDE

(35) DtμCp2fDE(x,t)=K∂x2p2fDE(x,t).

We point out that ${\hat{M}}_{2}\left(s\right)$ is not a completely Bernstein function (CBF) since its algebraic inverse is not a Stieltjes function (SF). Formally, we can take the inverse Laplace transform of Equation (33) with initial condition (2), but, due to the results given in Ref. [48], it will not be a well-posed Cauchy problem. Hence, we limit our consideration only to the Laplace space in which the solution of Equation (33) can be written as

$${\hat{p}}_{2}\left(x,s\right)={\left[{\hat{M}}_{2}\left(s\right)\right]}^{-1}\hat{N}\left(x,s/{\hat{M}}_{2}\left(s\right)\right).$$

The MSD corresponding to $p_{2}\left(x,t\right)$ becomes

$${\langle {\hat{x}}^{2}\left(s\right)\rangle }_{2}=\frac{1}{{\hat{M}}_{2}\left(s\right)}\;{\langle {\hat{x}}^{2}\left(s/{\hat{M}}_{2}\left(s\right)\right)\rangle }_{N}=\frac{2K}{\tau }\frac{s^{-\mu -1}}{s+\tau^{-1}},$$

from which, by inverse Laplace transform, one finds [5] (Equation (24)), namely

(36) $$\begin{array}{c} {\langle x^{2}\left(t\right)\rangle }_{2}=2K\tau^{\mu }{\left(t/\tau \right)}^{1+\mu }E_{1,2+\mu }\left(-t/\tau \right). \end{array}$$

In the case of stochastic resetting, we express the renewal equation given by Equation (8) for j=2 in Laplace space as

$${\hat{p}}_{r,2}\left(x,s|x_{0}\right)=\frac{s+r}{s}{\hat{p}}_{2}\left(x,s+r\right)=\frac{s+r}{s}\frac{1}{{\hat{M}}_{2}\left(s+r\right)}\hat{N}\left(x,\frac{s+r}{{\hat{M}}_{2}\left(s+r\right)}\right),$$

which, in the long-time limit, approaches the NESS equal to

(37) $$\begin{array}{cc} p_{r,2}^{\mathrm{st}}\left(x\right) & =\frac{r}{{\hat{M}}_{2}\left(r\right)}\hat{N}\left(x,\frac{r}{{\hat{M}}_{2}\left(r\right)}\right)=\frac{1}{2}\sqrt{\frac{r^{\mu }\left(r\tau +1\right)}{K}}\exp\left(-\sqrt{\frac{r^{\mu }\left(r\tau +1\right)}{K}}\left|x-x_{0}\right|\right). \end{array}$$

Therefore, the NESS is given by the Laplace distribution, as well, with a stationary variance $\sigma_{2}^{\mathrm{st}}=K/\left[r^{\mu }\left(r\tau +1\right)\right]$ around the average value $x_{0}$. For μ=1, NESS reduces to the one for the standard telegrapher’s process under resetting [8]. For τ=0, it turns to the NESS for an anomalous diffusion process under resetting [24,30,31,32,33].

The corresponding MSD in the case of resetting becomes

$${\langle {\hat{x}}^{2}\left(s\right)\rangle }_{r,2}=\frac{2K}{\tau }s^{-1}\frac{{\left(s+r\right)}^{-\mu }}{\left(s+r\right)+\tau^{-1}},$$

that is

(38) $${\langle x^{2}\left(t\right)\rangle }_{r,2}=2K\tau^{\mu -1}\int_{0}^{t}e^{-rt^{\prime }}{\left(\frac{t^{\prime }}{\tau }\right)}^{\mu }E_{1,1+\mu }\left(-\frac{t^{\prime }}{\tau }\right)dt^{\prime }.$$

From here, for the short-time limit, we have the behavior ${\langle x^{2}\left(t\right)\rangle }_{r,2}∼t^{1+\mu }$, while for the long-time limit we observe saturation

$${\langle x^{2}\left(t\right)\rangle }_{r,2}=\frac{2Kr^{-\mu }}{r\tau +1}.$$

**Figure 4:**
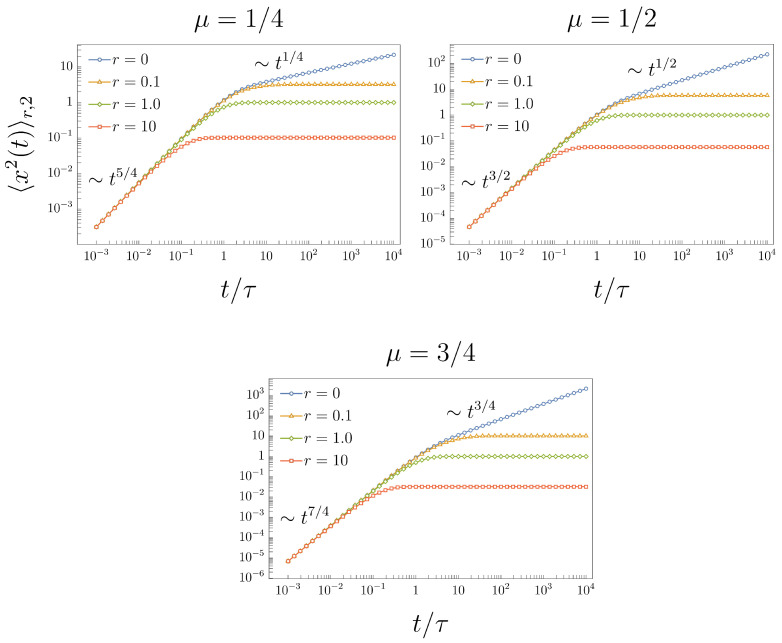
MSD (38) under the effects of stochastic resetting for different values of μ and the resetting rate, r={0, 0.1, 1.0, 10.0}. The case r=0 corresponds to the MSD (36).

This crossover dynamics of the MSD (38) for different values of μ and *r* is shown in Figure 4.

### First-Passage Time Problem

We can first solve the backward equation for the survival probability without resetting and then directly find the survival probability with resetting. It reads

τDt1+μCQ2(x0,t)+DtμCQ2(x0,t)=K∂x2Q2(x0,t),

with the same initial and boundary conditions as before, we find the survival probability

$${\hat{Q}}_{2}\left(x_{0},s\right)=\frac{1}{s}\left[1-\exp\left(-\sqrt{\frac{r^{\mu }\left(\tau r+1\right)}{K}}x_{0}\right)\right],$$

from where for the first-passage time density we have

$${\hat{P}}_{2}\left(s\right)=1-s{\hat{Q}}_{2}\left(x_{0},s\right)=\exp\left(-\sqrt{\frac{s^{\mu }\left(\tau s+1\right)}{K}}x_{0}\right).$$

The efficiency then becomes

(39) $$\begin{array}{c} \begin{array}{cc} E_{2} & =\int_{0}^{\infty }\exp\left(-\sqrt{\frac{s^{\mu }\left(\tau s+1\right)}{K}}x_{0}\right)ds\\ \; & =\frac{1}{\mu \tau \sqrt{\pi }}{\left(\frac{4K}{x_{0}^{2}}\right)}^{2}H_{2,1}^{1,2}\left[{\left(\frac{4K\tau^{\mu }}{x_{0}^{2}}\right)}^{\frac{1}{\mu }}\left|\begin{array}{c} \left[-\frac{3}{2},\frac{1}{\mu }\right],\left[2,1+\frac{1}{\mu }\right]\\ \left[1,1\right] \end{array}\right]\right., \end{array} \end{array}$$

where $H_{p,q}^{m,n}$ is the Fox *H* function in which the argument is given by ${\left[\left(4K\tau^{\mu }\right)/x_{0}^{2}\right]}^{1/\mu }$, and the upper and lower list of parameters reads [−3/2,1/μ], [2,1+1/μ], and [1,1], respectively. The derivation of the lower formula in Equation (39) is presented in Appendix D.

In the presence of resetting, we can directly find the survival probability from the corresponding one without resetting, from where for the MFPT we obtain

(40) $$T_{2,r}\left(x_{0}\right)=\frac{1}{r}\left[\exp\left(\sqrt{\frac{r^{\mu }\left(\tau r+1\right)}{K}}x_{0}-1\right)\right].$$

For μ=1, we obtain the known result for the MFPT in the case of a standard telegraph process. We can find the optimal resetting rate *r* for which MFPT is minimal, i.e.,

$$\partial_{r}T_{2,r}\left(x_{0}\right){|}_{r=r_{*}}=0,$$

from where we have

(41) $$\frac{\mu +r_{*}\tau +\mu r_{*}\tau }{2(r_{*}\tau +1)}\sqrt{\frac{r_{*}^{\mu }\left(\tau r_{*}+1\right)}{K}}x_{0}=1-\exp\left(-\sqrt{\frac{r_{*}^{\mu }\left(\tau r_{*}+1\right)}{K}}x_{0}\right),$$

i.e.,

$$1-e^{-\xi_{2}}=r\frac{d\xi_{2}}{dr},\;\xi_{2}=\sqrt{\frac{r_{*}^{\mu }\left(\tau r_{*}+1\right)}{K}}x_{0}.$$

**Figure 5:**
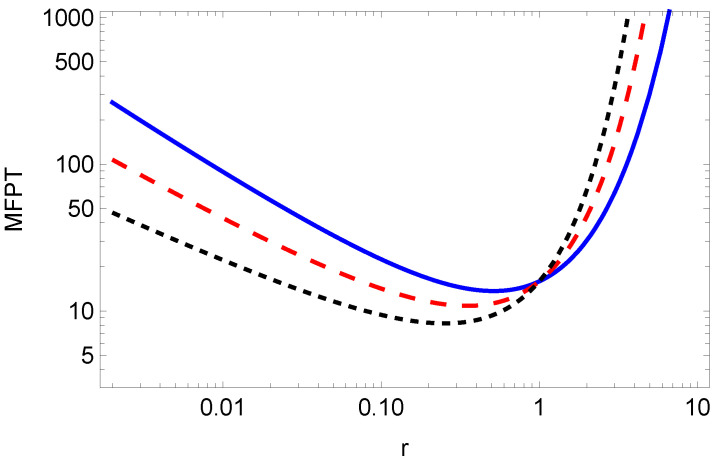
MFPT (40) for $x_{0}=2$, τ=1, K=1, μ=1/2 (blue solid line), μ=3/4 (red dashed line), μ=1 (black dotted line).

**Figure 6:**
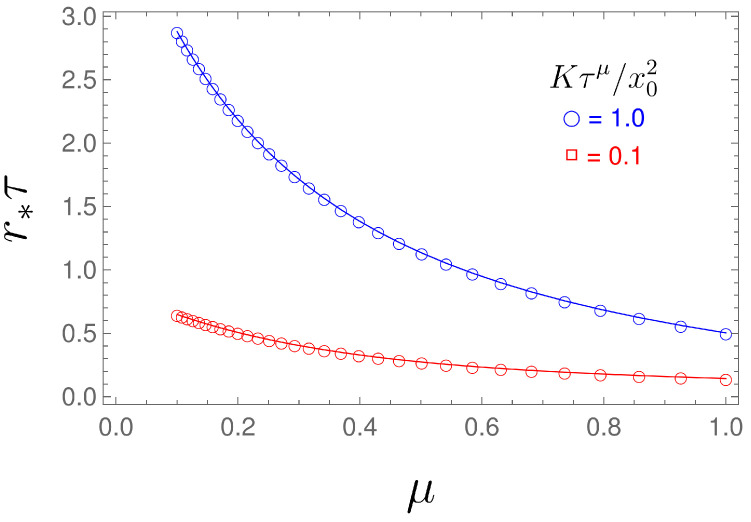
Optimal resetting rate $r_{*}$ versus μ, obtained by numerically solving Equation (41).

The dependence of the MFPT on the resetting rate is shown in Figure 5, while the changing of the optimal resetting rate by changing parameter μ is demonstrated in Figure 6. We see that by increasing parameter μ, the optimal resetting rate decreases, the behavior which was also observed for the FTE-I.

## 4. Summary

In this paper, we considered two distinct kinds of fractional telegrapher equations. In the absence of resetting, we found that both generalizations of the telegrapher equations describe fractional-ballistic transport in the short-time regime, and transit to fractional diffusion transport in the long-time one. FTE-I can be obtained in two ways based on the integral decomposition method, both ways lead to the same results. The methods consist of presenting the solution for the FTE-I from either the normal distribution or from the PDF of the telegraph process. The solution of FTE-II is presented only by use of the normal distribution in the integral decomposition.

Further, we analyzed these two fractional telegraphic processes in the presence of Poissonian resetting, which means that after a random time drawn from an exponential distribution, the particle is reset to the initial position. We found that in the long-time limit, due to the resetting in both cases, the particle reaches non-equilibrium stationary states while the MSDs saturate. In both cases, if the resetting rate is large enough, the nonequilibrium stationary distribution is determined by the short-time regime of the solution of the corresponding FTE, while if the resetting rate is small, the stationary distribution is determined by the long-time regime of the corresponding solution of the FTE.

We also considered the first-passage time problem, and for both cases, we calculated the survival probability, efficiency, and MFPT. It is shown that there is an optimal resetting rate for which the MFPT is minimal. This optimal resetting rate depends on the anomalous diffusion parameter μ. Additionally, the efficiency in the first case decreases faster with μ than in the second case.

Analysis of the generalized telegraph processes under non-instantaneous resetting [49,50,51], partial resetting [52,53], and resetting in an interval [54,55,56] are left for future investigation. The first-passage time problem could also be of interest in the case of resetting to multiple [57] and random positions [58]. Finding the connection between the Shannon and Fisher functionals [59] could also be of interest to future research.

## Figures and Tables

**Figure A1:**
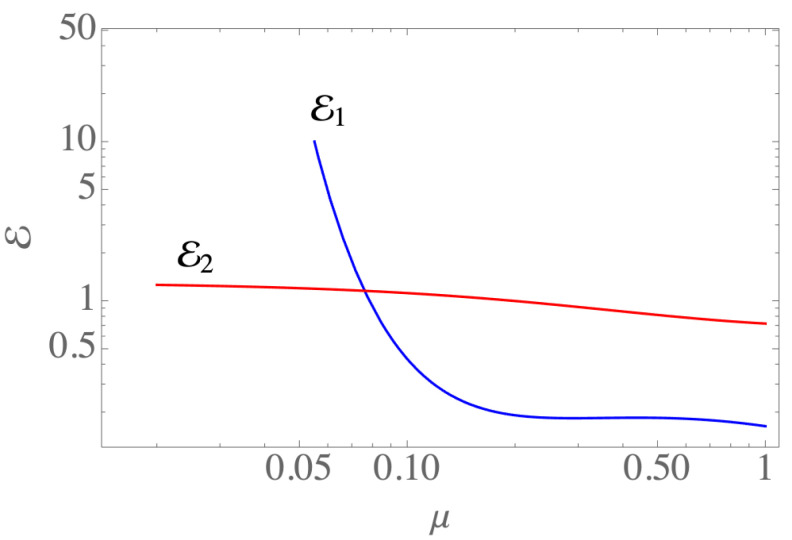
Comparison of the efficiencies for FTE-I and FTE-II from Equations (30) and (39), for K=1, $x_{0}=2$, τ=1.

## Data Availability

No new data were created or analyzed in this study. Data sharing is not applicable to this article.

## Abbreviations

The following abbreviations are used in this manuscript:

TE	Telegrapher’s Equation
FTE	Fractional Telegrapher’s Equation
MSD	Mean Squared Displacement
MFPT	Mean First-Passage Time

## Appendix A. Fractional Calculus and Mittag–Leffler Functions

The fractional derivative in the Caputo sense reads

DtνCf(t)=∫0tf(n)(ξ)(t−ξ)ν+1−ndξΓ(n−ν),ν∈(n−1,n),n∈N.

The three-parameter Mittag–Leffler function is defined by [60]

(A1) $$\begin{array}{c} E_{\rho ,\beta }^{\delta }\left(z\right)=\sum_{n=0}^{\infty }\frac{{\left(\delta \right)}_{n}}{\Gamma (\rho n+\beta )}\frac{z^{n}}{n!}, \end{array}$$

Its Laplace transform reads

$$L\left[t^{\beta -1}E_{\rho ,\beta }^{\delta }\left(-\lambda t^{\rho }\right)\right]=\frac{s^{\rho \delta -\beta }}{{\left(s^{\rho }+\lambda \right)}^{\delta }},$$

where $|\lambda /s^{\rho }|<1$. Note that for δ=1, it becomes the two parameter Mittag–Leffler function, $E_{\rho ,\beta }^{1}\left(z\right)=E_{\rho ,\beta }\left(z\right)$, and for β=δ=1, it becomes the one parameter Mittag–Leffler function, $E_{\rho ,1}^{1}\left(z\right)=E_{\rho ,1}\left(z\right)=E_{\rho }\left(z\right)$.

The Fox *H* function is defined through the Mellin transform as follows

(A2) Hp,qm,nz|[ap,Ap][bq,Bq]:=∫γL∏i=1mΓ(bi+Bis)∏i=1nΓ(1−ai−Ais)∏i=n+1pΓ(ai+Ais)∏i=m+1qΓ(1−bi−Bis)x−sds2πi,

where the parameters are subject to conditions

$$\begin{array}{c} \begin{array}{cc} \; & z\neq 0,\;0\leq m\leq q,\;0\leq n\leq p;\\ \; & a_{i}\in C,\;\;\;A_{i}>0,\;i=1,\ldots ,p;\;b_{i}\in C,\;\;\;B_{i}>0,\;i=1,\ldots ,q;\\ \; & \left[a_{p},A_{p}\right]=\left(a_{1},A_{1}\right),\ldots ,\left(a_{p},A_{p}\right);\;\left[b_{q},B_{q}\right]=\left(b_{1},B_{1}\right),\ldots ,\left(b_{q},B_{q}\right);\\ \; & \left(a_{p}\right)=a_{1},a_{2},\ldots ,a_{p};\;\left(b_{q}\right)=b_{1},b_{2},\ldots ,b_{q}. \end{array} \end{array}$$

## Appendix B. Completely Monotone and Bernstein Functions

Among the non-negative functions, we can distinguish the complete monotone and Bernstein functions. Based on [29], their definitions and subclasses are given below.

A function $\hat{c}:\left(0,\infty \right)\mapsto R$ is a *completely monotone function (CMF)* if its derivatives exist and ${\left(-1\right)}^{n}{\hat{c}}^{\left(n\right)}\left(s\right)\geq 0$, for all $n\in N_{0}$.

The important feature of CMF is that the Bernstein theorem uniquely connects it with a non-negative function c(t) through the Laplace integral

$$\hat{c}\left(s\right)=\int_{0}^{\infty }e^{-st}c\left(t\right)dt.$$

A (non-negative) *Stieltjes function (SF)* is a function $\hat{g}:\left(0,\infty \right)\mapsto \left[0,\infty \right)$ which can be written as

$$\hat{g}\left(s\right)=\frac{A}{s}+B+\int_{0}^{\infty }\frac{\sigma (dt)}{s+t},$$

where A,B≥0 and σ is a measure on (0,∞) such that $\int_{0}^{\infty }\sigma \left(dt\right)/\left(1+t\right)<\infty$. Note that the class of SFs belongs to the class of CMFs, and thus, it is a decreasing function. Usually, it is assumed that A=0 and B=0, which simplifies the calculations and ensures its integrability at zero and vanishing at infinity.

A function $\hat{b}:\left(0,\infty \right)\mapsto R$ is a *Bernstein function (BF)* if its all derivatives exist, $\hat{b}\left(s\right)$ is non-negative, ${\hat{b}}^{\prime }\left(s\right)$ is a CMF. If a BF $\hat{b}:\left(0,\infty \right)\mapsto \left(0,\infty \right)$, and, additionally, $\hat{b}\left(s\right)/s$ is an SF, then it is called a *completely Bernstein function (CBF)*.

The characteristics of these functions and their properties can be referenced in [29]. The key features highlighted in this paper include (i) the product or sum of CMFs results in another CMF; (ii) the composition of CMF and BF(CBF) yields a CMF; (iii) for the CBF, there exists a corresponding partner ${\hat{b}}^{★}$ such that $\hat{b}\left(s\right){\hat{b}}^{★}\left(s\right)=s$; and (iv) the algebraic inverse of SF (CBF) is CBF (SF).

## Appendix C. Efros Theorem

The Efros theorem [61,62,63,64,65] generalizes the convolution theorem for the Laplace transform. It states the following:

**Theorem** **A1.** If $\hat{G}\left(s\right)$ and $\hat{q}\left(s\right)$ are analytic functions, and

$$L\left[g\left(x,\xi \right);s\right]=\hat{g}\left(x,s\right)$$

as well as

$$L\left[f\left(\xi ,t\right);s\right]=\int_{0}^{\infty }f\left(\xi ,t\right)e^{-st}dt=\hat{G}\left(s\right)e^{-\xi \hat{q}\left(s\right)},$$

exist, then

$$\hat{G}\left(s\right)\hat{g}\left(x,\hat{q}\left(s\right)\right)=\int_{0}^{\infty }\;\left[\;\int_{0}^{\infty }\;\;g\left(x,\xi \right)f\left(\xi ,t\right)d\xi \right]e^{-st}dt.$$

From Efros theorem appears that

(A3) $$L^{-1}\left[\hat{G}\left(s\right)\hat{g}\left(x,\hat{q}\left(s\right)\right);t\right]=\int_{0}^{\infty }g\left(x,\xi \right)f\left(\xi ,t\right)d\xi .$$

## Appendix D. Derivation of the Efficiencies $E_{1}$ and $E_{2}$

The derivation of $E_{1}$ and $E_{2}$ base on using the Laplace transform of the Lévy–Smirnov distribution $\Phi_{1/2}\left(\sigma \right)=\exp\left[-1/\left(4\sigma \right)\right]/\left(2\sqrt{\pi }\sigma^{3/2}\right)$, this is

$$\begin{array}{c} \begin{array}{cc} e^{-az^{1/2}} & =\int_{0}^{\infty }dt\;e^{-zt}a^{-2}\Phi_{1/2}\left(ta^{-2}\right)\\ \; & =\int_{0}^{\infty }dt\;e^{-zt}\frac{a}{2\sqrt{\pi }t^{3/2}}e^{-a^{2}/\left(4t\right)}. \end{array} \end{array}$$

Inserting it into Equation (30) with $z=\tau^{\mu }s^{2\mu }+s^{\mu }$ and into Equation (39) with $z=\tau s^{\mu +1}+s^{\mu }$, and next changing the order of integrals we get

(A4) $$E_{j}=\int_{0}^{\infty }dt\;\frac{x_{0}}{2\sqrt{\pi K}t^{3/2}}e^{-x_{0}^{2}/\left(4Kt\right)}{\mathrm{Int}}_{j},\;j=1,2.$$

The auxiliary integral ${\mathrm{Int}}_{j}$ for the efficiencies $E_{j}$ respectively read

$${\mathrm{Int}}_{1}=\int_{0}^{\infty }ds\;e^{-t\tau^{\mu }s^{2\mu }-ts^{\mu }}\;\mathrm{and}\;{\mathrm{Int}}_{2}=\int_{0}^{\infty }ds\;e^{-t\tau s^{\mu +1}-ts^{\mu }}.$$

At first, we calculate ${\mathrm{Int}}_{1}$ by setting $s^{2\mu }=y$, which can be expressed as

(A5) $$\begin{array}{c} \begin{array}{cc} {\mathrm{Int}}_{1} & =\frac{1}{2\mu }\int_{0}^{\infty }y^{\frac{1}{2\mu }-1}e^{-t\tau^{\mu }y-ty^{1/2}}\\ \; & =\frac{1}{\mu }\Gamma \left(1+\frac{1}{2\mu }\right)e^{t/(8\tau^{\mu })}D_{-1/\mu }\left(\sqrt{\frac{t}{2\tau^{\mu }}}\right), \end{array} \end{array}$$

where we used [66] (Equation (2.2.1.16)). The parabolic cylinder function is standard denoted as $D_{-1/\mu }\left(\cdot \right)$. Then, we substitute Equation (A5) into Equation (A4) and change $t^{1/2}$ onto ξ. That leads to

$$E_{1}=\frac{x_{0}}{\mu \sqrt{\pi K}2^{1/\mu }}\Gamma \left(1+\frac{1}{2\mu }\right)\int_{0}^{\infty }d\xi \;\xi^{-\frac{1}{\mu }-2}e^{-\frac{x_{0}^{2}}{4K}\xi^{-2}+\frac{\xi^{2}}{8\tau^{\mu }}}D_{-1/\mu }\left(\frac{\xi }{\sqrt{2\tau^{\mu }}}\right),$$

which, after using [67] (Equation (2.11.4.4)), gives the lower formula in Equation (30).

Proceeding analogically, we derive the explicit form of $E_{2}$. Now, setting $s^{1/(1+\mu )}=y$, we can present ${\mathrm{Int}}_{2}$ as

$${\mathrm{Int}}_{2}=\frac{1}{1+\mu }\int_{0}^{\infty }dy\;y^{-\frac{\mu }{1+\mu }}e^{-t\tau y-ty^{\mu /(1+\mu )}}.$$

This integral can be calculated for fractional μ=l/k, such that l<k by employing [66] (Equation (2.2.1.22)) and then transforming the results into the more transparent language of the Fox *H* function according to formula [68] (Equation (8.3.2.22)). That gives

Int2=1μ(tτ)−11+μH1,11,1τt1/μ[1,1+1μ][11+μ,1],

which substitutes into Equation (A4) for j=2 and after changing $t^{-1}$ onto ξ yields

E2=x02μKπτ−11+μ∫0∞ξ32+11+μe−x024KξH1,11,1τξ1/μ[1,1+1μ][11+μ,1]dξ=1μτπ4Kx0224Kτμτx0211+μH2,11,24Kτμx021/μ[−32−11+μ,1μ],[1,1+1μ][11+μ,1],

where we used [68] (Equation (2.25.2.3)). Next, applying [68] (Equation (8.3.1.8)) allows us to get the lower formula in Equation (39).

A comparison of the expressions for the efficiencies in each case as a function of μ is shown in Figure A1. In the FTE-I case, the efficiency decreases with μ, ranging from high values for small μ to low values for μ close to one. In contrast, in the FTE-II case, although efficiency decreases with μ, it is maintained close to 1 for the whole range of values.
